# A picture is worth a thousand words: using digital tools to visualise marine invertebrate diversity data along the coasts of Mozambique and São Tomé & Príncipe

**DOI:** 10.3897/BDJ.9.e68817

**Published:** 2021-09-24

**Authors:** Henrique Niza, Marta Bento, Luis F. Lopes, Alexandra Cartaxana, Alexandra M. Correia

**Affiliations:** 1 Faculdade de Ciências da Universidade de Lisboa, Lisboa, Portugal Faculdade de Ciências da Universidade de Lisboa Lisboa Portugal; 2 MARE - ULisboa, Lisboa, Portugal MARE - ULisboa Lisboa Portugal; 3 Instituto de Investigação Científica e Tropical (IHMT) and Global Health and Tropical Medicine (GHTM), Lisboa, Portugal Instituto de Investigação Científica e Tropical (IHMT) and Global Health and Tropical Medicine (GHTM) Lisboa Portugal; 4 Centre for Ecology, Evolution and Environmental Changes (cE3c), Faculdade de Ciências da Universidade de Lisboa, Lisboa, Portugal Centre for Ecology, Evolution and Environmental Changes (cE3c), Faculdade de Ciências da Universidade de Lisboa Lisboa Portugal; 5 Museu Nacional de História Natural e da Ciência (MNHNC), Lisboa, Portugal Museu Nacional de História Natural e da Ciência (MNHNC) Lisboa Portugal

**Keywords:** biodiversity, coastal marine fauna, macroinvertebrates, Mozambique channel, online mapping, São Tomé and Príncipe

## Abstract

The amount of biological data available in online repositories is increasing at an exponential rate. However, data on marine invertebrate biodiversity resources from Mozambique and São Tomé and Príncipe are still sparse and scattered. Online repositories are useful instruments for biodiversity research, as they provide a fast access to data from different sources. The use of interactive platforms comprising web mapping are becoming more important, not only for the scientific community, but also for conservation managers, decision-makers and the general public as they allow data presentation in simple and understandable visual schemes. The main goal of this study was to create an interactive online digital map (hosted and available at MARINBIODIV Atlas), through the collection of data from various sources, to visualise marine invertebrate occurrences and distribution across different habitats, namely mangroves, seagrasses, corals and other coastal areas, in Mozambique and São Tomé and Príncipe. The acquired biodiversity data were managed and structured to be displayed as spatial data and to be disseminated using the geographic information system ArcGIS, where data can be accessed, filtered and mapped. The ArcGIS web mapping design tools were used to produce interactive maps to visualise marine invertebrate diversity information along the coasts of Mozambique and São Tomé and Príncipe, through different habitats, offering the foundation for analysing species incidence and allocation information. Understanding the spatial occurrences and distribution of marine invertebrates in both countries can provide a valuable baseline, regarding information and trends on their coastal marine biodiversity.

## Introduction

There is an exponential increase in the amount of biological data available in online repositories. In biodiversity studies, digital repositories are useful resources because they provide centralisation of available global knowledge, enable prompt accessibility, incorporate data from multiple sources around the world, allow more holistic data analysis and accurate reproducible studies (Maldonado et al. 2015). Digital biodiversity repositories have been continuously growing and data are often submitted in the form of large datasets such as global or regional species occurrence lists. These large databases are not exempt from errors, inaccuracies and omissions, such as taxonomic uncertainties and geographical inaccuracies of species occurrences (Hortal et al. 2015). In spite of this, these repositories are extremely useful, providing uniformed data from a number of sources that greatly exceed what could be gathered manually, thereby saving time, money and reducing the impact of more in situ sampling on biodiversity (Edwards 2004, Guralnick and Hill 2009, Chapman 2015). In fact, there has been a growing standardisation and availability of biodiversity data in online repositories, enabling quick access to expand canonical data from different origins. The Global Biodiversity Information Facility (GBIF at www.gbif.org), which promotes the publishing of datasets using generally agreed data standards on biodiversity, is one of these repositories. Other online repositories are accessible and complement each other, such as the Integrated Digitised Biocollections (iDigBio at www.idigbio.org), citizen contribution-based systems like iNaturalist (at http://www.inaturalist.org) and Biodiversity4all (at www.biodiversity4all.org). Beyond "big" data, biodiversity repositories such as Natural History Collections (NHC) are significant scientific infrastructures with valuable data on the biodiversity of the planet since they contain curated sets of natural objects that are collected over time, in different locations, with associated relevant information digitised or in paper (Cartaxana et al. 2014). Other data sources on biodiversity include scientific articles and checklists, either digital or paper-based, often resulting from more in-depth studies. Therefore, the compilation and incorporation of biodiversity data dispersed through a variety of different sources into spatial explicit digital formats is also a significant step in making information accessible to a wide range of purposes and audiences (Asaad et al. 2019).

Maps are suitable tools to communicate complex spatial information, being extremely useful to explore contents and for raising awareness about different issues. For instance, maps on species occurrences and spatial patterns are mandatory tools to provide biodiversity information for environmental resource management. The increase of georeferenced species occurrence data enables the use of geographic information system (GIS) tools that can be applied for geographic data representations through, for example, the creation of accurate distribution maps. High quality, robust and consistent data and information on species occurrences at different spatial and temporal levels, allow the use of GIS to manage digital biodiversity data from various sources to analyse it and display it in a spatially explicit manner (Wahid et al. 2016, Wright 2016). The advantage of GIS is that it models reality based on data, as it is designed to capture, model, store, receive, share, manipulate, analyse and present geographically referenced information (ESRI 1990). Basic GIS operations now provide a secure basis for measuring, mapping and analysing data. Data stored in a GIS database provides a simplified version of the Earth's surface. Georeferenced data can be organised by a GIS using different criteria, for example, thematic maps or spatial objects. Each thematic layer can be saved using an appropriate data format, depending on its nature and the purpose of its use. GIS are key in determining priority for species taxonomic identification and conservation, historical mapping to analyse trends and planning the spatial use of resources. It also serves as an integral component in the spatial modelling of species distribution in the past and in possible future scenarios (Worboys and Duckham 2004).

For greater accessibility, web mapping is the method of using interactive maps made accessible on the internet by GIS. These may implement filters that allow the user to choose the data to be displayed, deriving different levels of information. For the scientific community, the public and policy-makers, the use of interactive platforms consisting of web maps is becoming increasingly important, as they allow up-to-date data to be presented using clear and understandable visual systems (Cristofori et al. 2015, Cristofori et al. 2017, Demarchi et al. 2017, Vincent et al. 2018). By using different and collaborative mapping software, such as free-to-use Google Maps and Bing Maps, open-source QGIS and OpenStreetMap or cloud-based ArcGIS, it is possible to create web maps. They allow maps to be generated and have several functions, to view and interact with maps and geospatial data (Sui 2014). As biodiversity and habitat loss rates increase, it is crucial that we develop a simpler and more effective way of incorporating all biodiversity data into interactive digital platforms, such as web maps and encourage the open sharing of data, so that scientists, analysts and policy-makers can apply it for research and policy decisions (Rocha et al. 2014). Since web mapping has been an area of strong growth in the last decade, the result of this expansion is the number of biodiversity projects that use this methodology to graphically display the data (Veenendaal et al. 2017). Projects aimed at mapping biodiversity at specific locations, such as China (Lin et al. 2018), Japan (NIES and JBIF 2015), Kansas (Kansas Biological Survey 2020) and the Coral Triangle (Asaad et al. 2019); on specific taxonomic groups as in the case of ants (Janicki et al. 2016); or on unique characteristics, such as invasive or disease-related species monitoring (NAS - Nonindigenous Aquatic Species 2020 and Mosquito Alert 2020, respectively), are increasingly popular.

The growth of human populations within coastal areas has increased due to rural-urban migration, with people relocating to more urbanised and economic centres. This migration increases human pressure on the environment due to land and marine-based human activities. As a result, coastal and marine living resources and their habitats are being adversely lost or damaged, reducing marine biodiversity (Celliers and Ntombela 2015). Nearshore habitats are of great socio-economic significance, especially in sub-Saharan Africa. For instance, Mozambique's and São Tomé and Príncipe's coastal populations depend on marine resources to sustain their livelihoods and food security (Vicente and Bandeira 2014). Marine invertebrates comprise important food sources for local populations, especially for the poorest people who depend on these resources for their livelihoods and food security and may have high commercial, gastronomic and ecological importance (Paula and Silva 1998, Anderson 2009). However, data on resources related to marine invertebrate biodiversity in these countries are still scarce and dispersed. Therefore, aggregating this information, thus bringing it into practical application, is of high importance.

The main objectives of this study were to: 1) integrate comprehensive data on marine invertebrates from mangroves, seagrasses, corals and other coastal areas of Mozambique (MOZ) and São Tomé and Príncipe (STP) into an interactive GIS mapping system and 2) disseminate this information online through the web mapping MARine INvertebrate BIODIVersity (MARINBIODIV Atlas) along the coasts of Mozambique and São Tomé and Príncipe. We explored existing digital records of marine biodiversity from MOZ and STP to generate species occurrence distribution maps and made these available online through a web map - MARINBIODIV Atlas. These data increased our understanding of marine invertebrate biodiversity along the coasts of MOZ and STP contributing with baseline information on coastal marine invertebrate occurrences and distribution in both countries. Further, the MARINBIODIV Atlas provides a new tool for science, policy-making and legislating, as well as for engaging Mozambican and São Tomé and Príncipe’s citizens with science and the preservation of their natural resources.

## Material and methods

**Figure 1.**
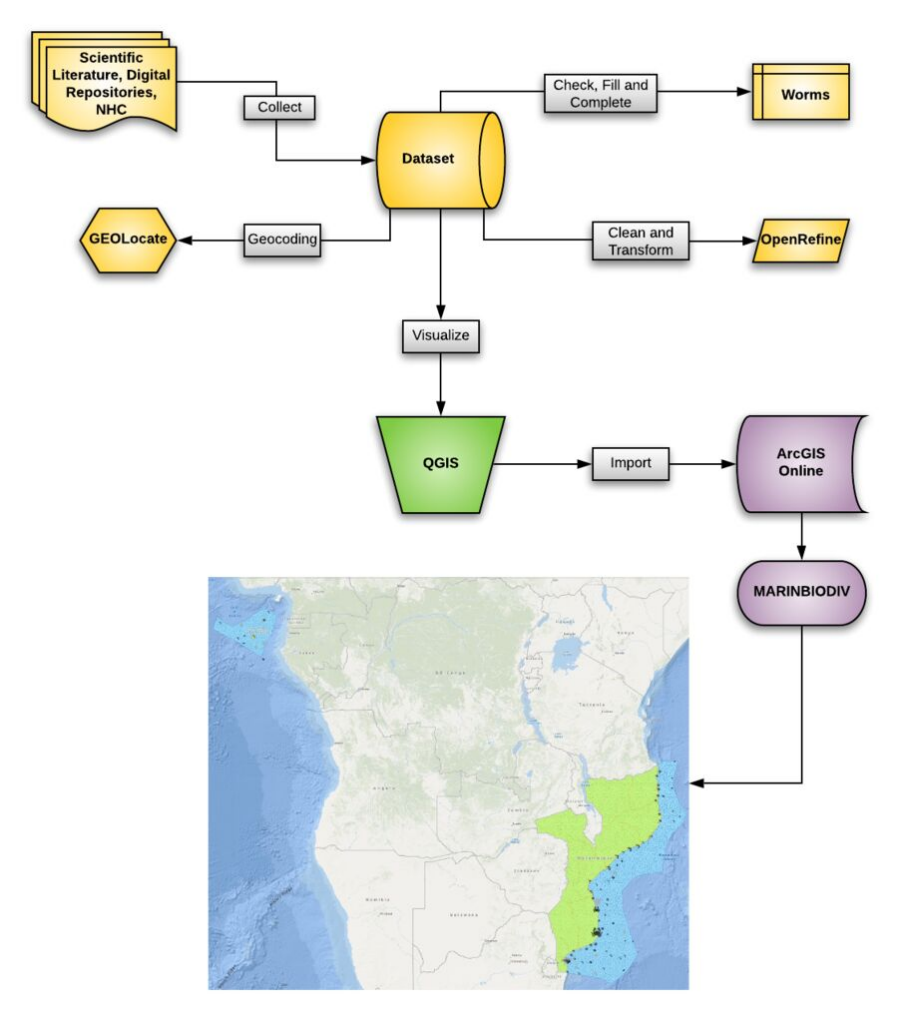
Workflow of the study depicting the main steps used to construct the interactive web map: data collection in yellow, data representation in green and data dissemination in purple (created using the Lucidchart web-based application).

This study comprised the use of digital tools: (1) to create an interactive geographic data representation of marine invertebrate species occurrences and distribution and respective habitats, across the coastal zones of MOZ and STP in ArcGIS Desktop (ArcMap 10.7.1), by a comprehensive compilation of biodiversity data contained in digital repositories, NHC records and scientific literature and (2) to construct an interactive digital platform map (MARINBIODIV Atlas) for online dissemination using ArcGIS Online, specifically designed for web mapping (Fig. 1).

### Geospatial Data Representation

The biodiversity database was created by combining and organising data from MOZ and STP on marine invertebrates, as well as aggregating global biodiversity data from digital repositories. Annelida, Arthropoda, Cnidaria, Echinodermata and Mollusca were chosen as the phyla that were most representative of the study areas and habitats. Specifically, data were gathered from worldwide open-source information from online digital biodiversity repositories, such as GBIF (Suppl. material 1) and iDigBio, NHC records from worldwide museums and scientific literature. Data were first organised, cleaned up and validated in a Microsoft Excel spreadsheet because of its simplicity. In the Excel spreadsheet, each line corresponded to a single occurrence, i.e. an observation or sampling in a defined geographic location and period. Only the occurrences with taxon rank equal to genus, species and subspecies were considered. Data were catalogued into a Darwin Core (DwC) metadata schema-based structure (Darwin Core maintenance group 2014), collated, geocoded and validated and then imported to an ArcGIS database. A large percentage of the data collected did not have geographic coordinates. Therefore, geocoding, verification and correction of geographic coordinates were accomplished using the GEOLocate Collaborative Georeferencing Web Client interface (GeoLocate Developer Resources 2019). Records with the general description of “Off” (e.g. Zambeze River, Off Mouth) were geocoded 200 to 300 metres in diameter from the locality. The uncertainty of the records was dismissed. Any records unable to geocode with GEOLocate, were either discarded or manually searched and georeferenced using Google Maps. Since data originated from multiple sources, it was necessary to make it uniform, to ensure data standardisation for reliable and high standards. The data were cleaned and wrangled using the open-source desktop application OpenRefine v.3.1 and the taxonomic names were validated using the WoRMS checklist (WoRMS Editorial Board 2019).

Geographic analysis, using QGIS, entailed steps, such as geographical data processing and merging different habitat layers. Habitat data collected from images instead were georeferenced using the inbuilt QGIS *Georeferencer* function. In this case, the georeferencing process – which involves taking a raster image coverage, assigning a coordinate system and coordinates to it and translating, transforming and warping it into a position relative to some other spatial data – was accomplished by assigning real-world coordinates to specific pixels on the raster obtained by the coordinates on the map image itself.

**Figure 2.**
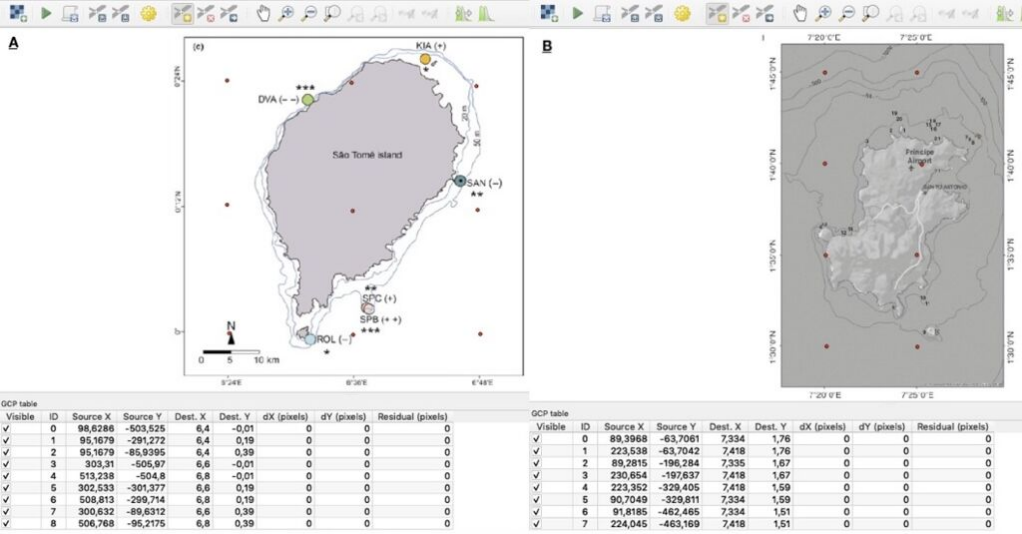
Using the QGIS Georeferencer interface, two raster datasets from literature are used to identify Mangrove habitats: **A.** the coloured circles and **B.** the numbers. Red dots indicate raster Ground Control Points.

For georeferencing, a total of nine ground control points were used in the raster relative to São Tomé Island and eight ground control points for the raster relative to Príncipe Island (Fig. 2).

The habitats studied encompassed mangroves, seagrasses and corals present in the coastal zones of MOZ and STP. The spatial datasets mapping the coastal habitats, added as layers, were downloaded from the UN Environment World Conservation Monitoring Centre website at http://data.unep-wcmc.org and the ReefBase website at http://reefbase.org/gis_maps/datasets.aspx. The datasets, used for each habitat, were as follows: Coral - Global Distribution of Coral Reefs (Dataset ID: WCMC-008): the dataset shows the global distribution of coral reefs in tropical and subtropical regions, composed of one set of polygon occurrence data, with a temporal range from 1954 to 2018 and the reference system WGS 1984 (version 4.0 - November 2018); Coral Bleaching (Dataset: ReefBase): the dataset provides point occurrence data of observation details of coral bleaching around the world, with a temporal scope since early 2002; Monitoring Sites (Dataset: ReefBase): the dataset provides point occurrence data on coral reef monitoring sites locations from major reef monitoring programmes. Reefs Location (Dataset: ReefBase): the dataset provides point occurrence data on coral reef locations; Marine Protected Areas (Dataset: ReefBase): the dataset provides point occurrence data on marine protected areas with coral reef zones. Mangrove: World Atlas of Mangroves (Dataset ID: WCMC-011): the dataset shows the global distribution of mangroves and it was produced mostly from satellite imagery, composed of one set of polygon occurrence data, with a temporal series mainly from 1999 to 2003 and the reference system WGS 1984 (version 2.0 – December 2017); Global Distribution of Mangroves USGS (Dataset ID: WCMC 010): the dataset shows the global distribution of mangrove forests derived from earth observation satellite imagery, composed of one set of polygon occurrence data, with a temporal range from 1997 to 2000 and the reference system WGS 1984 (version 1.3 – June 2015); Global Mangrove Watch (Dataset ID: GMW-001): the dataset shows a global baseline map of mangroves using satellite imagery, composed of one set of polygon occurrence data, with a temporal array from 1996 to 2016. Data retrieved on 4 April 2019 (version 2.0). Seagrass: Global Distribution of Seagrasses (Dataset ID: WCMC-013-014): the dataset shows the global distribution of seagrasses, composed of two subsets of point and polygon occurrence data, with a temporal range from 1934 to 2015 and the reference system WGS 1984 (version 6.0 - June 2018). The search was expanded to the scientific literature to resolve the lack of habitat information in São Tomé and Príncipe.

The layers with the same geometry type, for example, "Point" or "Polygon," were merged into a single layer using the command "Merge Vector Layers" to combine all data corresponding to each habitat (corals, mangroves and seagrasses) in a single shapefile.

The process of vectorisation generated several thousands of small polygons in some places, which created overlapping polygons. To correct these, a dissolve operation was performed with Mapshaper software. (Spalding et al. 2010, Giri et al. 2011, UNEP-WCMC and FT 2017, Bunting et al. 2018, UNEP-WCMC et al. 2018).

The input layers “Global Distribution of Coral Reefs”, “Coral Bleaching”, “Monitoring Sites”, “Reefs Location” and “Marine Protected Areas” were merged into a point data layer named “Coral point-data”. Both input layers “Global Distribution of Mangroves USGS” and “Global Mangrove Watch” were merged into a polygon data layer named “Mangrove polygon-data”. All layers created manually were also joined to their respective habitat layers. Region layers were downloaded from public domain map data available online: administrative boundaries, divisions and outline of MOZ and STP as ESRI Shape file format latitude and longitude coordinates at GADM data website at https://gadm.org/data.html; Mozambican and São Tomé and Príncipe EEZ as Shapefile format at Marine Regions website at www.marineregions.org.

### Online Data Dissemination

The data were imported to ArcMap as a CSV file with latitude and longitude coordinates stored in separated columns. Point coordinates’ longitude and latitude were mapped to X and Y fields, respectively. The coordinate reference system used was EPSG:4326 or WGS 1984. The ArcMap layouts are specifically designed to provide a foundation for web mapping species occurrences and distribution data across MOZ and STP habitats. Based on point data and/or polygon data, the arrangement of combined data corresponding to the three habitat layers (corals, mangroves and seagrass) provides the basis for the filtering of habitat types.

To promote online data dissemination and make it user-friendly, a digital platform web map (MARINBIODIV Atlas) was developed to visualise marine invertebrate diversity along the coasts of Mozambique and São Tomé and Príncipe, by using the complete cloud-based ArcGIS mapping software, ArcGIS Online, designed for web mapping and exploring data through filtering and mapping different layers of information.

## Results

**Figure 3.**
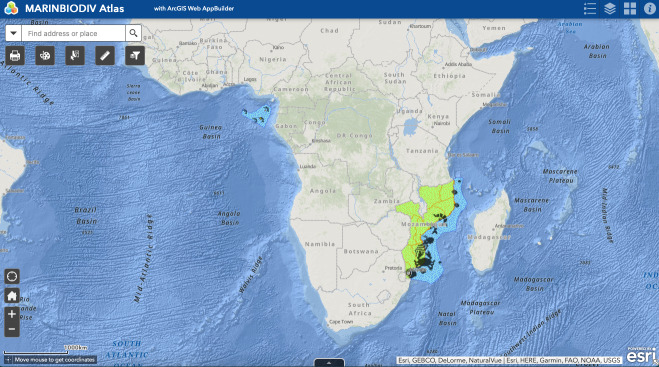
The default scale/zoom of MARINBIODIV Atlas.

**Figure 4.**
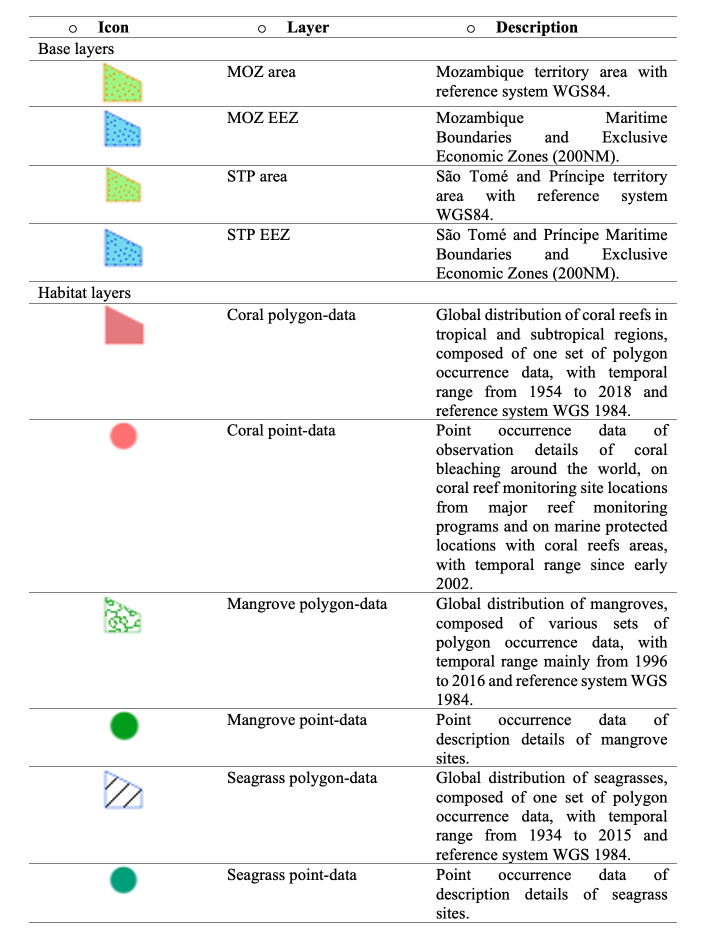
MARINBIODIV Atlas layers list.

MARINBIODIV Atlas web map is an interactive digital platform that can be used to visualise the occurrences and distribution of invertebrate species along the coastlines of MOZ and STP. It provides a variety of filter layers to manipulate the data, allowing the visualisation of occurrences against specific criteria (e.g. type of habitat, taxonomic classification, amongst others). The web map contains 11 layers that can be selected or unselected to filter the data in display. These layers are grouped in three main sub-groups: 1) species occurrences, 2) habitats and 3) MOZ and STP boundaries. To provide geographical context, the continents and oceans are also represented in the background (Figs 3, 4).

**Figure 5.**
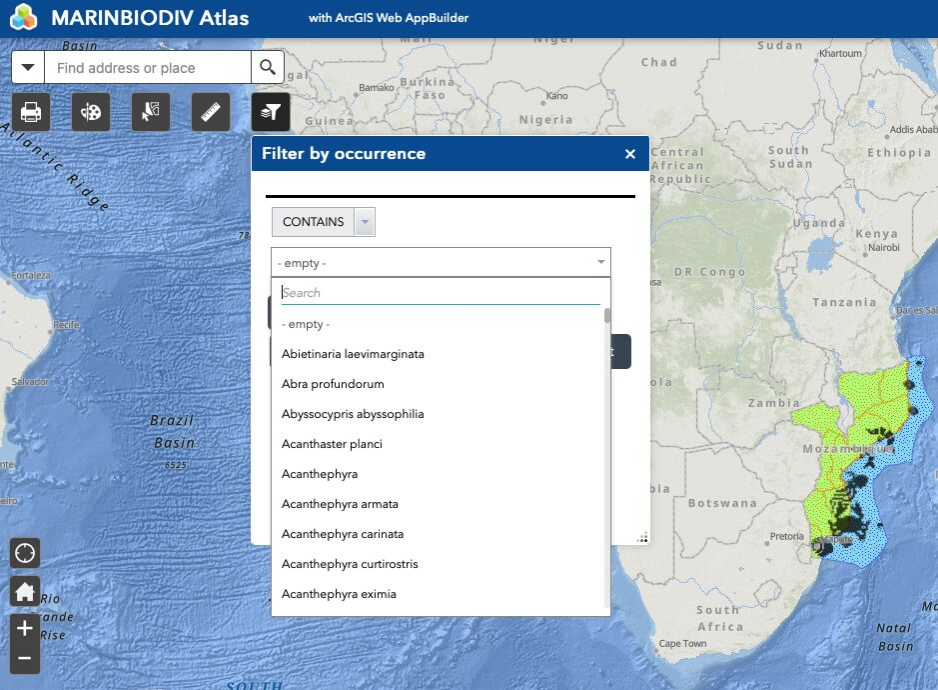
Representation of the filter by occurrences widget on the map.

**Figure 6.**
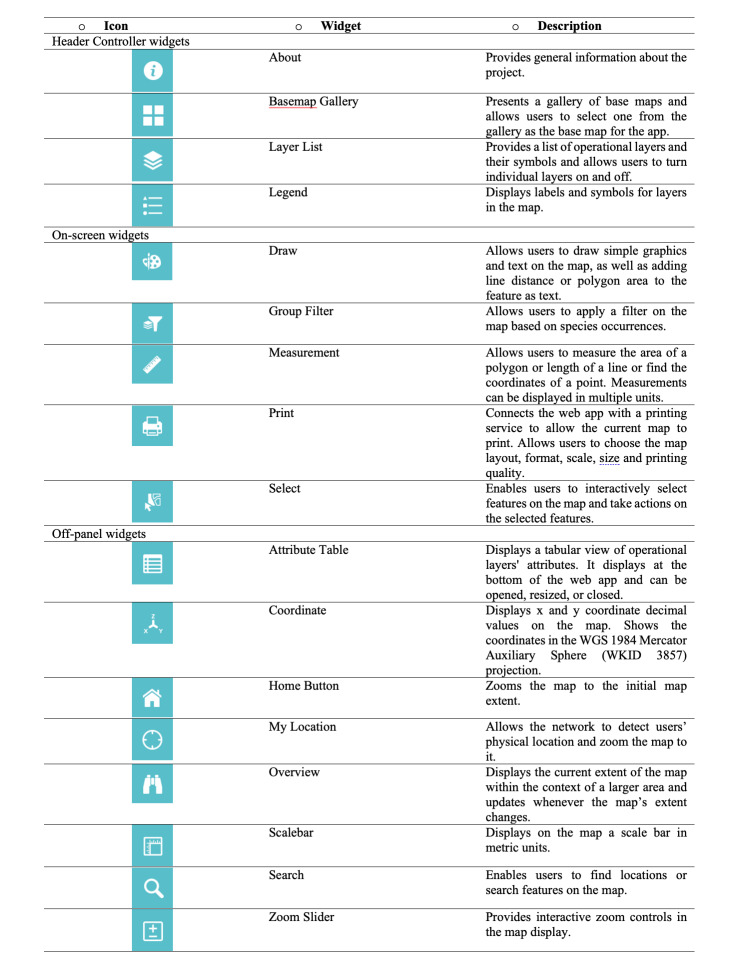
MARINBIODIV Atlas widgets list.

The web map's homepage uses a full-screen canvas template, presenting part of Africa, as well as the Atlantic and Indian Oceans comprising the study areas. Filtering can be done through the collapsible layers' menu, at the top right side of the map, which includes five layers (species occurrences, MOZ and STP areas and EEZ). The occurrences in the map are clustered, i.e. symbols scale proportionally to the number of occurrences of a given species at a location. Species, genus or family can be searched through the filter symbol at the top left of the map (Fig. 5). See Fig. 6 for all widgets of this web map.

**Figure 7.**
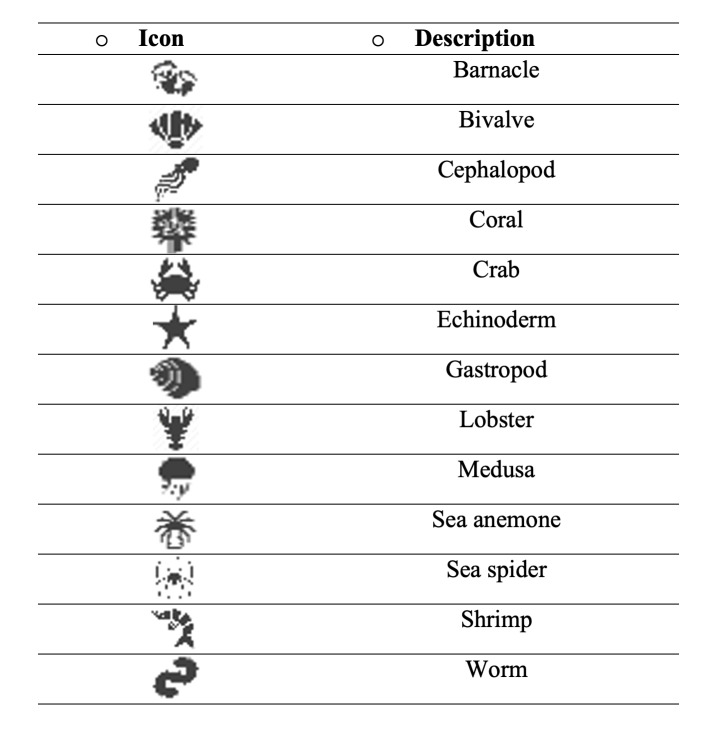
MARINBIODIV Atlas species occurrences legend.

**Figure 8.**
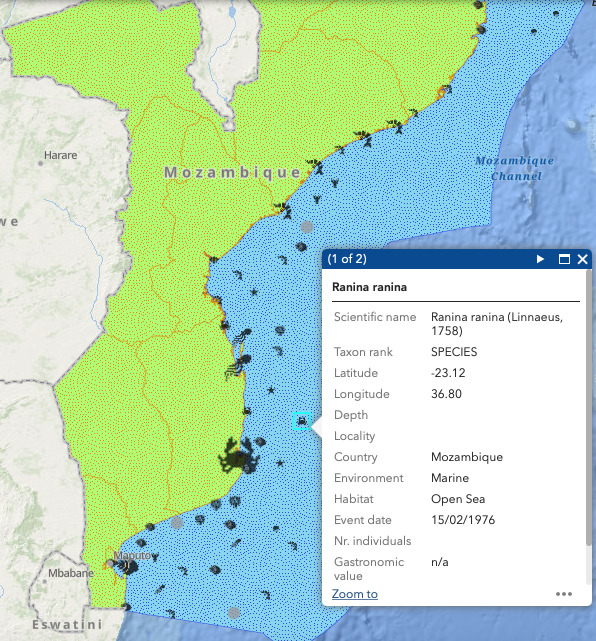
Zoom in on the Mozambique portion of the web map to show the various symbols, based on the specimen's typology and the information box.

The species occurrence layers are separated into 13 main classes, represented by specific symbols: barnacle, bivalve, cephalopod, coral, crab, echinoderm, gastropod, lobster, medusa, sea anemone, sea spider, shrimp and worm (Fig. 7). Each class includes, respectively: 11 species, 190 species, 27 species, 167 species, 205 species, 176 species, 686 species, 75 species, 56 species, 6 species, 5 species, 362 species and 45 species. These icons do not correspond to single species, but rather to morphotypes, i.e. groups of species that have a similar shape within a broader taxonomic group. Symbols vary in size according to the number of individuals per occurrence (by using the proportional symbol scale as referred above). By hovering over each symbol, an information box is displayed with details and statistics on each occurrence, such as scientific name, taxon rank, latitude, longitude, depth, locality, country, environment, habitat, event date, numbers of individuals, gastronomical value and an external link to the WoRMS website for general information (Fig. 8).

**Figure 9.**
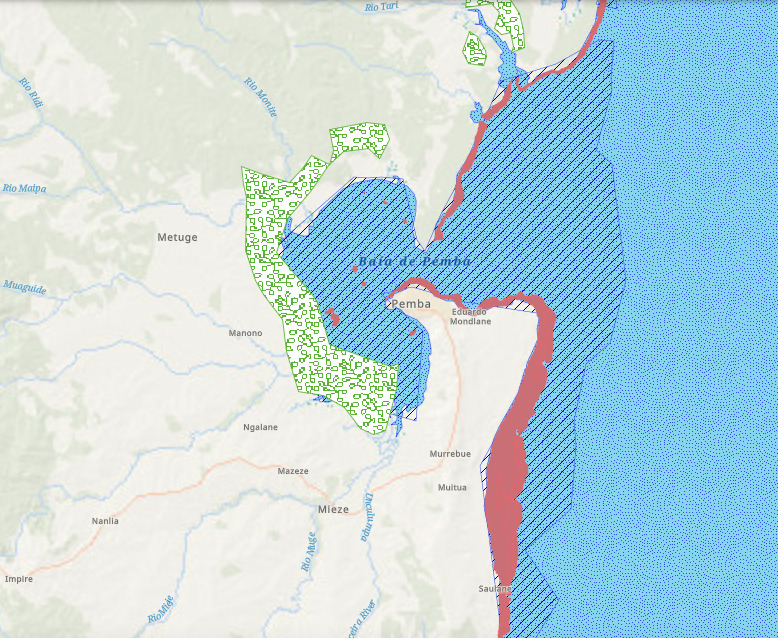
Types of habitats present along the coast of Mozambique that are represented in MARINBIODIV Atlas as polygon-data: corals in red, mangroves in green and seagrasses in blue.

**Figure 10.**
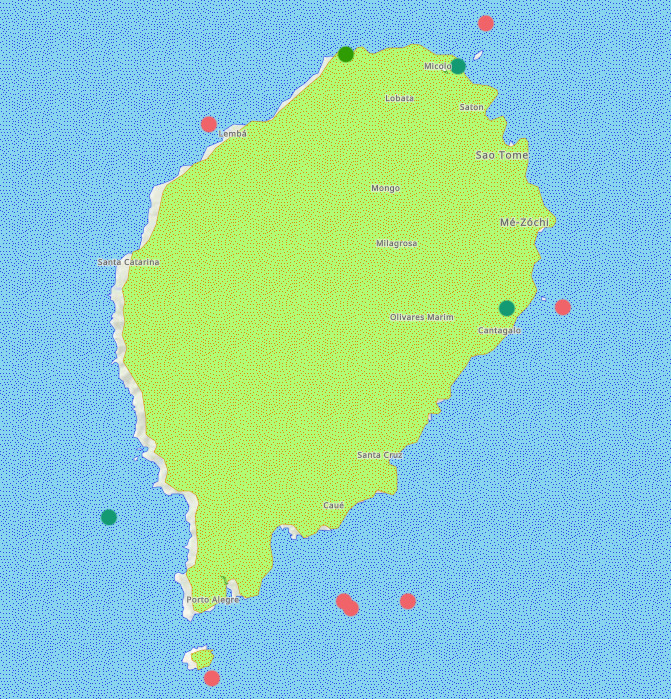
Types of habitats present along the coast of São Tomé and Príncipe are depicted as point-data in the Web Map: corals in red, mangroves in green and seagrasses in blue.

Habitats are divided into three groups: corals, mangroves and seagrasses, with polygon-data and point-data layers, each represented with specific symbology (Figs 9, 10). They can be merged and/or seen individually.

**Figure 11.**
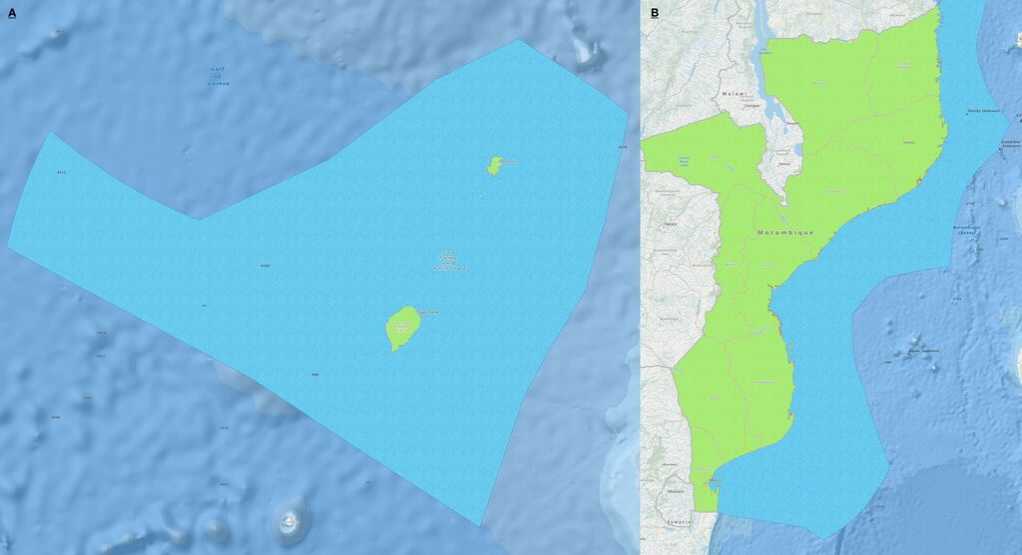
Representation of the boundaries of **A.** São Tomé and Príncipe and **B.** Mozambique.

Fig. 11 represents the administrative boundaries of both STP and MOZ, including their names, outlining their borders and exclusive economic zones.

The interactive digital platform is hosted and available at MARINBIODIV Atlas.

## Discussion

Marine biodiversity is essential to human well-being providing essential services, such as nutrient cycling, ecosystem stability, food, medicinal resources and recreation, amongst others. Thus, it is of the utmost importance to gather existing knowledge and transmit it to decision-makers so that governments, together with the civil society, safeguard biodiversity health. We compiled and integrated data on marine invertebrates from mangroves, seagrasses and corals along the coastal zones of Mozambique and São Tome and Príncipe. These data were incorporated into a web platform to assemble an interactive map, MARINBIODIV Atlas, on the occurrence and distribution of marine invertebrates across different habitats in MOZ and STP, to disseminate and share the obtained information with the scientific community, conservation managers, policy-makers and the general public. As biodiversity loss continues and limited resources are available to preserve and protect biodiversity, replication of this type of approach in other regions and other species (e.g. fishes) is important (Janicki et al. 2016, Asaad et al. 2019).

MARINBIODIV Atlas was developed using the ArcGIS Online software, which allowed the creation and combination of multiple habitat layers, as well as other information layers and to define marker symbology. One of the major challenges for the development of this Atlas was related to the preparation of habitat shapefiles, compiled from multiple, varying scale and quality data sources. While some used consistent methodology across all regions, others were less consistent, including observational data from different regional, national and international sources. These factors generated a mismatch in the position of the layers in relation to the coastline, which was corrected as far as possible, by creating representative polygons, based on satellite imagery. Overall, most polygons used in this work are relatively well spatially aligned to the coastline layer. In spite of our best efforts to reduce spatial representation bias, accuracy may vary amongst locations because layer sources were different and related errors were not consistent across datasets, including cloud cover, background noise, Landsat scanline error and misclassification of certain areas due to striping artifacts, amongst others. Nevertheless, precision is best measured on the seaward side when compared to the landward side due to the presence of terrestrial vegetation (Asaad et al. 2019). Additionally, using zero or low code tools, such as ArcGIS Online, for web mapping can be very useful, avoiding much work on writing complex code. This platform has been used for the development of a myriad of maps presenting spatial data about environment, habitat and species occurrences, compiled from the largest biodiversity datasets in their respective fields (NIES and JBIF 2015, Lin et al. 2018, Asaad et al. 2019, Kansas Biological Survey 2020).

Habitat mapping is an effective method to gain a better understanding of biodiversity in a given region. Mangroves, seagrasses and corals were the only habitats mapped along the MOZ and STP coastlines. The lack of other mapped habitat types may generate limitations to fully assess the ecological and biological significance of these marine regions. To avert these constraints, data from “open sea” and “other coastal areas” were also included in MARINBIODIV Atlas. Data gaps related to habitat mapping might be explained by a lack of research financing and geopolitical instability. These generally hinder data collection and monitoring programmes aimed at improving representation and understanding of those countries' biodiversity and ecosystems, resulting in less available information (Kassas 2002). Since marine protected areas can have dramatically different ecological features and varied habitats, integrated habitat mapping throughout time, as we have done here, can reveal a wealth of information about how development has harmed different habitats and if it is continuing to do so. It can also help to obtain a detailed view of the biological significance of each habitat, as well as the species that they maintain (Pomeroy et al. 2004). Furthermore, by being spatially explicit, maps convey simple tools that may help communities to construct on less environmentally desirable territory. In fact, habitat maps provide knowledge that local governments can use for a variety of purposes, including land use planning, conservation management, public awareness, habitat development and preservation (Smith et al. 2011).

Marine invertebrates are a major component of marine habitats, encompassing a highly diverse group (Tittensor et al. 2010). The MARINBIODIV Atlas enables a dataset of curated marine invertebrate biodiversity data to be accessed and visualised through a web browser with detailed geographical and taxonomic coverage. The integration of data on marine invertebrates from a variety of formats presented several hurdles. Collecting data from different sources, curating and processing these data, as well as associated digital resources, such as images, as well as geospatial reference information acquisition and manipulation, represented a challenge which was overcome. This web map provides different filter layers, allowing the visualisation of occurrences and distribution against specific criteria (e.g. type of habitat) and integrates data that otherwise would be scattered, heterogeneous and might be difficult to access depending on its source, hampering its contribution to biodiversity conservation (Flemons et al. 2007, Map of Life 2020). Species lists can be read at various points of interest for conservation and fisheries, controlling and centralising all biodiversity data, while other online maps are available for a single species (e.g. Fishbase, EOL) or multiple species (e.g. GBIF, iNaturalist) without the opportunity to view the entire listing. This map provides stakeholders with options for obtaining easily accessible accurate data and facilitates successful decision-making processes, as well as the ability of scientific communities to develop geospatial tools to support marine biodiversity conservation.

Nearshore habitats are extremely important socioeconomically, particularly in the western Indian Ocean, because 65 million people live within 10 kilometres of the coast in the greater Indian Ocean region (Burke et al. 2011). As a result, coastal and marine living resources, as well as their ecosystems, are being degraded or lost, resulting in a reduction in marine biodiversity. These pressures, which are more prevalent in developing countries, will disrupt biotic communities and ecosystem processes, putting biodiversity and local communities at risk. Marine invertebrates, such as decapod crustaceans, are essential food supplies for local populations, particularly the poorest, who rely on these resources for survival and food security (Garcia et al. 2019). All spatial data on marine invertebrates, from different coastal habitats of MOZ and STP, provided by the MARINBIODIV Atlas, contribute to the United Nations (UN) sustainable development goals (SDGs), namely SDG #14: “Life below water” referring to marine and coastal biodiversity, its conservation and sustainable use for human society's sustainable growth (United Nations Development Programme 2019a). This information is also relevant, as it can be related to the natural and gastronomic resources and food security in these two countries, adding on to SDG # 2: "No hunger”, aimed at ending hunger by achieving food security and improving nutrition worldwide (United Nations Development Programme 2019b). Furthermore, the small-scale fisheries sector contributes to the Voluntary Guidelines for Securing Sustainable Small-Scale Fisheries in the Context of Food Security and Poverty Eradication since it is deeply anchored in MOZ and STP local communities, traditions and values (Food and Agriculture Organization of the United Nations 2018).

By being freely accessible, the MARINBIODIV Atlas can be further used to develop new research projects, to create teaching or dissemination tools, to write books, articles and brochures for outreach, amongst other work programmes. The significance of this study lies in its ability to provide clear baseline biodiversity data and digital resources that can be used to model the distribution of marine invertebrate species and estimate the size of species ranges in mangroves, seagrasses and corals along the coasts of Mozambique and São Tomé and Príncipe in order to predict extinction risk and, hopefully, to advance biodiversity conservation strategies.

## Conclusions

Due to an overwhelming and continual increase in data available online, integration of biodiversity data from multiple sources, formatted according to international standards, is vital for data analysis and critical for extracting knowledge for the field of biological sciences (Torres et al. 2006). We developed the MARINBIODIV Atlas, an interactive map with data on the occurrence and distribution of marine invertebrates across mangroves, seagrasses and corals from Mozambique and São Tome and Príncipe, that provides an useful tool for research, education and to raise public awareness on the importance of marine macroinvertebrates and their habitats.

Further development of this study could have broader implications, such as providing a framework or baseline information for more detailed ecological research, resulting in the identification of natural areas and ecological networks to provide information for habitat preservation and restoration, strategic land-use planning, as well as marine invertebrate monitoring, management and conservation.

## Supplementary Material

9D4CABE5-7BB6-5F42-8E55-B967496B3695 10.3897/BDJ.9.e68817.suppl1 Supplementary material 1 GBIF-mediated occurrence data Data type occurrences Brief description Mozambique and São Tomé and Príncipe occurrence data File: oo_556379.docx https://binary.pensoft.net/file/556379 GBIF
