# Supplementary material for: A picture is worth a thousand words: using digital tools to visualise marine invertebrate diversity data along the coasts of Mozambique and São Tomé & Príncipe
Source: Biodivers Data J. 2021 Sep 24;9:e68817. doi: 10.3897/BDJ.9.e68817 (PMC8486733; doi:10.3897/BDJ.9.e68817)
Supplement: Supplementary material 1 — GBIF-mediated occurrence data [file bdj-09-e68817-s001.docx]

**Mozambique occurrence data:**

Annelida:

GBIF.org (25 October 2018) GBIF Occurrence Download <https://doi.org/10.15468/dl.cgskgn>

Arthropoda:

GBIF.org (25 October 2018) GBIF Occurrence Download <https://doi.org/10.15468/dl.rsvrwu>

Decapoda MUHNAC:

GBIF.org (03 December 2018) GBIF Occurrence Download <https://doi.org/10.15468/dl.wtl0gh>

Cnidaria:

GBIF.org (25 October 2018) GBIF Occurrence Download <https://doi.org/10.15468/dl.tdo85o>

Echinodermata:

GBIF.org (25 October 2018) GBIF Occurrence Download <https://doi.org/10.15468/dl.ob3jcd>

Mollusca:

GBIF.org (25 October 2018) GBIF Occurrence Download <https://doi.org/10.15468/dl.uzkzcv>

**São Tomé and Príncipe occurrence data:**

Annelida:

GBIF.org (10 October 2018) GBIF Occurrence Download <https://doi.org/10.15468/dl.p7vvlf>

Arthropoda:

GBIF.org (10 October 2018) GBIF Occurrence Download <https://doi.org/10.15468/dl.q4jiv0>

Cnidaria:

GBIF.org (10 October 2018) GBIF Occurrence Download <https://doi.org/10.15468/dl.lrjhwj>

Echinodermata:

GBIF.org (10 October 2018) GBIF Occurrence Download <https://doi.org/10.15468/dl.isduid>

Mollusca:

GBIF.org (10 October 2018) GBIF Occurrence Download <https://doi.org/10.15468/dl.px6i6k>
